# Plantar keratoderma and curly hair as a diagnostic clue of cardiomyopathy risk

**DOI:** 10.1111/1346-8138.17192

**Published:** 2024-03-25

**Authors:** Stine Bjørn Gram, Klaus Brusgaard, Anette Bygum, Alex Hørby Christensen, Lilian Bomme Ousager

**Affiliations:** ^1^ Department of Clinical Genetics Odense University Hospital Odense Denmark; ^2^ Department of Clinical Research University of Southern Denmark Odense Denmark; ^3^ European Reference Network for Rare Skin Diseases (ERN‐Skin) Odense Denmark; ^4^ Hudklinikken Kolding Koldig Denmark; ^5^ Department of Cardiology Rigshospitalet and Herlev‐Gentofte Hospitals Hellerup Denmark

**Keywords:** cardiomyopathy, *DSP*, palmoplantar keratoderma

A 17‐year‐old woman visited the dermatology clinic due to thickened and painful skin on her soles since early childhood. Physical examination showed remarkable focal plantar keratoderma on pressure points on both feet (Figure 1a). Similar skin lesions were present in her mother, grandmother, and grandmother's sister. The patient and affected family members also had noticeable curly hair (Figure 1b). There was no known family history of heart disease. The patient was offered genetic testing, which revealed a likely pathogenic novel heterozygous frameshift variant in *DSP* (c.175dupA, p.Thr59Asnfs*34) and a diagnosis of hereditary palmoplantar keratoderma was confirmed. *DSP* encodes the desmoplakin protein, a component of desmosomes. Desmosomes are intercellular junctions that function to resist mechanical stress and present in both epidermal and myocardial tissue. *DSP* is associated with heterogeneous phenotypes comprising varying skin and hair abnormalities, as well as desmoplakin‐related heart disease, including arrhythmogenic and dilated cardiomyopathy, ventricular arrhythmias, and myocarditis‐like episodes.^1^, ^2^, ^3^ Cardiac examinations performed in the family members showed normal results at the time of evaluation. However, other variants predicted to have the same effect on protein function, including variants in the same exon, have been linked to cardiomyopathy.^4^, ^5^ Therefore, cardiac surveillance was offered.

**FIGURE 1:**
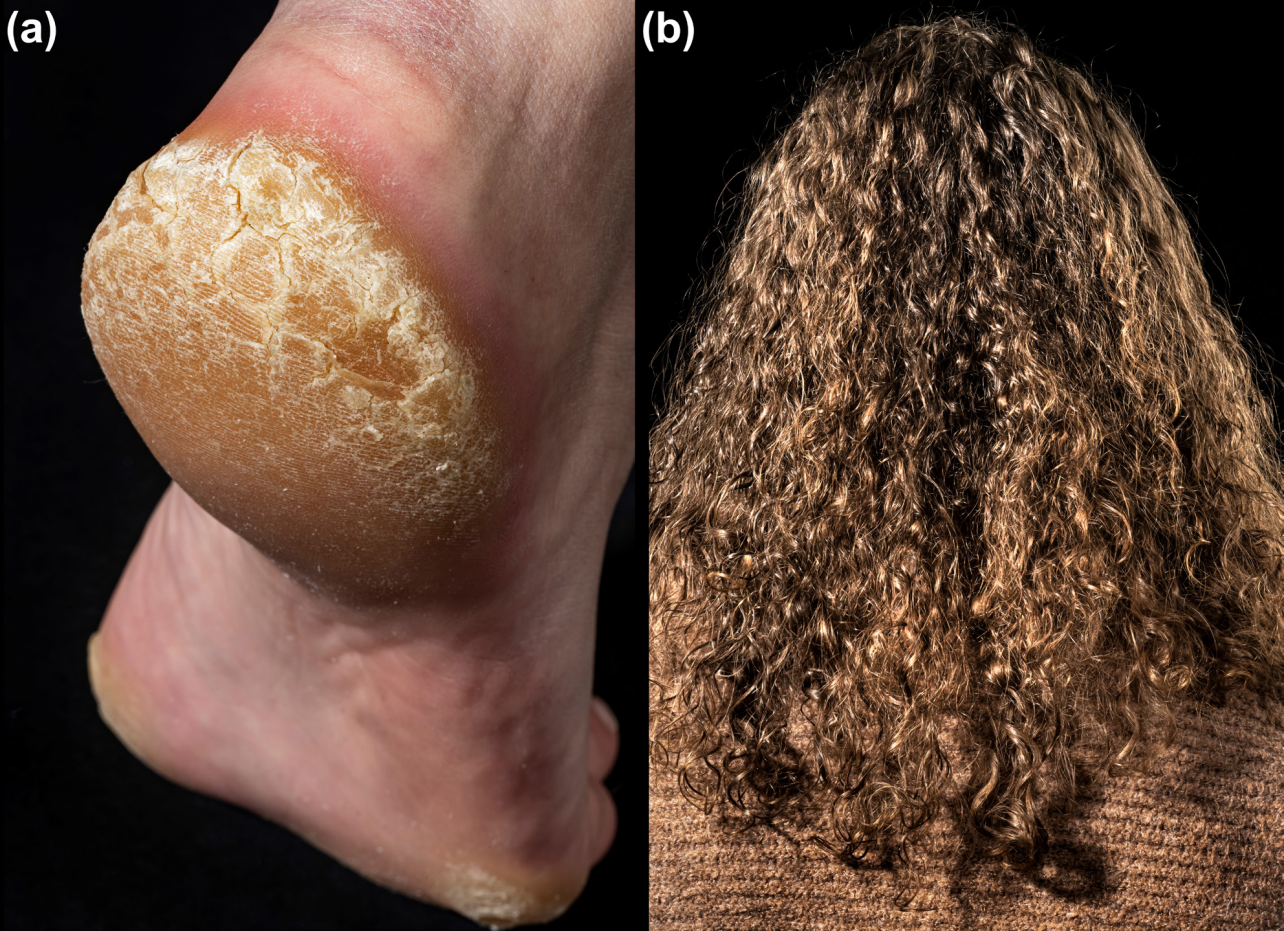
Clinical presentation showing (a) plantar keratoderma on pressure points and (b) curly hair.

The co‐occurrence of palmoplantar keratoderma and curly hair serves as an important diagnostic clue for the potential presence of an undiagnosed cardio‐cutaneous syndrome. Genetic testing can confirm the diagnosis of either an autosomal recessive or autosomal dominant inherited disease. The cardiac manifestations associated with *DSP* variants are still not fully understood and may not be uniformly present across all affected family members.^1^ However, sudden life‐threatening cardiac events may be the initial manifestation and cardiac evaluation of at‐risk individuals is crucial. Consequently, the presence of both palmoplantar keratoderma and curly hair serves as an unexpected, yet vital diagnostic indicator for a potential unidentified risk of heart disease.

## CONFLICT OF INTEREST STATEMENT

None declared.

## CONSENT

The authors obtained informed consent from the patient for publication of this report.
